# A Framework for Learning Analytics Using Commodity Wearable Devices

**DOI:** 10.3390/s17061382

**Published:** 2017-06-14

**Authors:** Yu Lu, Sen Zhang, Zhiqiang Zhang, Wendong Xiao, Shengquan Yu

**Affiliations:** 1 Advanced Innovation Center for Future Education, Beijing Normal University, Beijing 100875, China; yusq@bnu.edu.cn; 2 Institute for Infocomm Research, Agency for Science, Technology and Research, Singapore 138632, Singapore; 3 School of Automation and Electrical Engineering, University of Science and Technology, Beijing 100083, China; zhangsen@ustb.edu.cn (S.Z.); wdxiao@ustb.edu.cn (W.X.); 4 School of Electronic and Electrical Engineering, University of Leeds, Leeds LS29JT, UK; z.zhang3@leeds.ac.uk

**Keywords:** wearable sensors, learning analytics, pervasive computing, activity recognition

## Abstract

We advocate for and introduce LEARNSense, a framework for learning analytics using commodity wearable devices to capture learner’s physical actions and accordingly infer learner context (e.g., student activities and engagement status in class). Our work is motivated by the observations that: (a) the fine-grained individual-specific learner actions are crucial to understand learners and their context information; (b) sensor data available on the latest wearable devices (e.g., wrist-worn and eye wear devices) can effectively recognize learner actions and help to infer learner context information; (c) the commodity wearable devices that are widely available on the market can provide a hassle-free and non-intrusive solution. Following the above observations and under the proposed framework, we design and implement a sensor-based learner context collector running on the wearable devices. The latest data mining and sensor data processing techniques are employed to detect different types of learner actions and context information. Furthermore, we detail all of the above efforts by offering a novel and exemplary use case: it successfully provides the accurate detection of student actions and infers the student engagement states in class. The specifically designed learner context collector has been implemented on the commodity wrist-worn device. Based on the collected and inferred learner information, the novel intervention and incentivizing feedback are introduced into the system service. Finally, a comprehensive evaluation with the real-world experiments, surveys and interviews demonstrates the effectiveness and impact of the proposed framework and this use case. The F1 score for the student action classification tasks achieve 0.9, and the system can effectively differentiate the defined three learner states. Finally, the survey results show that the learners are satisfied with the use of our system (mean score of 3.7 with a standard deviation of 0.55).

## 1. Introduction

As a recently-emerging and fast-growing research field, learning analytics refers to “the measurement, collection, analysis and reporting of data about learners and their contexts” [1]. Driven by the data from heterogeneous resources and the latest data mining techniques, learning analytics mainly attempts to understand and model the learning process, which would eventually enable teachers and schools to tailor educational opportunities to individual student’s need [2] and provide effective interventions. During this process, recognizing the learner’s physical actions, typically including the curricular and extracurricular ones, is considered as a key research topic, as a fine-grained distinction and description of physical actions on the individual level would directly help to create a deeper and robust understanding of learners, in turn offering the holistic synthesis of their learning process. Moreover, the physical action information can serve as a significant clue to infer the so-called learner context, which refers to all of the learner’s physical and cognitive statuses related to the learning process (e.g., student physical and cognitive engagement level in class). However, this research topic remains under-supported, starting from the first step that collects and identifies the learner’s physical actions at a high granularity, until the last step that uses the physical actions to infer learner context and subsequently makes the necessary interventions. Furthermore, the entire process, from learner action identification until learner context analytics, is ideally hassle-free and non-intrusive to avoid unnecessary burdens on learners and their learning process.

On the other hand, the fast advances in sensor-based activity recognition techniques and the availability of commodity wearable devices greatly facilitate the capture of learner physical actions. The commodity wearable devices refer to the smart equipment that can be worn by users and purchased on the market, typically including wrist-worn devices (e.g., Apple smartwatch [3]) and eye wear devices (e.g., Google glass [4]). The built-in sensors, including accelerometer, gyroscope and magnetic sensors, have become an indispensable component on the commodity wearable devices and have been used to conduct human activity recognition [5]. Moreover, these wearable devices usually carry a multicore processor together with wireless communication interfaces (e.g., Bluetooth, Wi-Fi or 3G/4G modules) and, thus, provide a powerful platform to conduct data analytics for learner context inference and to establish the desired communication loop. In many cases, a wearable device can work together with its paired smartphone to accomplish more complex tasks and valuable services. Compared to the vision-based systems that can also capture learner physical actions (using single or multiple video cameras), wearable devices do not need any infrastructure to support and almost no maintenance cost. The commodity wearable devices are also different from the dedicated and customized wearable equipment, such as the smart textiles [6] or body sensor network [7], as they can be easily purchased from the market at a relatively inexpensive price. For example, in the exemplary use case of this work, the commodity smartwatch is less than 200 U.S. dollars each.

Motivated by the above analysis and observations for learning analytics, we propose and advocate for a novel framework, called LEARNSense, with two explicit design objectives: (1) effectively collect learner physical actions and learner context using commodity wearable device and sensor-based activity recognition techniques; (2) properly utilize the captured learner actions and context information to support services for optimizing the learning process. Furthermore, to provide the practical embodiments of the LEARNSense framework, we demonstrate an exemplary use case, which mainly conducts the in-class student physical action recognition and action-driven intervention tasks.

Our work in this paper principally makes the following key contributions:

- Novel framework for learning analytics: We propose a novel, but practical framework for learning analytics: it utilizes the fine-grained learner action sensing on the commodity wearable devices to provide learner context inference and build services for optimizing the learning process.
- Learner action and context sensing: We adopt a hierarchical decomposition approach to design and implement a sensor-based learner action and context information collector on commodity wearable devices. It is a hassle-free and non-intrusive solution, where the latest data mining and sensor data processing techniques are employed.
- Class engagement analyser: We demonstrate an exemplary use case specifically designed for the student class engagement analysis. The key innovations in this use case include: (i) the use of the inertial sensor data to accurately detect the four types of student physical actions in class; (ii) the use of the student physical actions to infer their engagement status; and (iii) the aggregation of the inferred engagement status to provide novel interventions and incentivizing feedback to students.

Besides the contributions listed above, we believe that the main impact of this work is to highlight the broader possibility of using learner physical action and context sensing results on commodity wearable devices to create innovative learning analytics services.

The rest of the paper is organized as follows: Section 2 gives the related work. Section 3 depicts the overall system architecture. Section 4 describes the learner context collector on wearable devices. In Section 5, we present the exemplary use case for the class engagement analysis, along with the empirical evaluation results. The discussion is given in Section 6. We finally conclude in Section 7.

## 2. Related Work

### 2.1. Learning Analytics

Taking advantage of the vast amounts of data generated from heterogeneous sources in the educational space, learning analytics [1] has become a fast-growing field in recent years, which mainly focuses on understanding and analysis of data generated during the learning process [8]. As part of these efforts, sensor data [9,10], online learning data [11] and teaching data [9] have been widely used to tackle a variety of problems and challenges for the learning analytics, including learner modelling [12], collaborative learning [13], teaching analytics [14] and privacy issues [15]. For example, Fidalgo et al. [16] utilize student online data to improve teamwork assessment; Guia et al. [17] use wearable device data to assist language learning for young children. From the perspective of data processing, multiple AI techniques, including data mining [18] and natural language processing [19], have been introduced to this field.

Among the previous studies above, reference [9] is most relevant to our work in terms of data type and activity recognition: it identifies teacher’s physical actions, such as explanation and questioning, using multiple wearable sensors (eye-tracking, EEG, accelerometer, audio and video) during classroom enactment. However, our work mainly focuses on the learner sides and does not rely on any specifically designed devices, but only the commodity wearable devices. Moreover, our exemplary use case shows that using only one type of commodity wearable device in tandem with the designed software application would be able to accurately capture the student actions and meanwhile infer class engagement status.

### 2.2. Wearable Technology and Activity Recognition

Wearable technology refers to the devices that can be worn by users, taking the form of an accessory. It has been regarded as an important development in educational technology, especially for K–12 education [20]. Grace et al. [21] propose to deploy a wearable computing platform for computing education. Lars et al. [22] employ wearable sensors to enable employees to reflect on experiences from their work. Many large corporations, such as Apple, Samsung and Google, already have a number of commodity smart wearable devices with difference functionalities, typically including sleep monitoring and fitness tracking. Adidas has deployed the specifically designed wrist-worn devices on school children for fitness tracking purposes [23]. Compared to the wearable devices, the traditional fixed-infrastructure systems, especially the video-based system, incur high maintenance costs over time. Moreover, the vision-based system has a low performance in natural lighting conditions, causing a shadowing of the points of interest [24]. In addition, the computational and storage costs for image processing are significantly higher than the sensor data analytics on the commodity wearable devices.

On the other hand, sensor-based activity recognition [5,25] has made rapid progress: the recent studies utilize novel features extracted from a variety of wearable sensors, especially the three-axis accelerometer, to recognize eating activities [26], shopping gestures [27] and social context [28]. Our activity recognition design falls into the same research field and adopts similar techniques. However, it innovates in recognizing the actions and context information specifically for learners and the learning environment.

Lastly, to the best of our knowledge, no previous work used the commodity wearable devices to conduct learner action recognition and context inference for learning analytics.

## 3. Framework Overview

**Figure 1:**
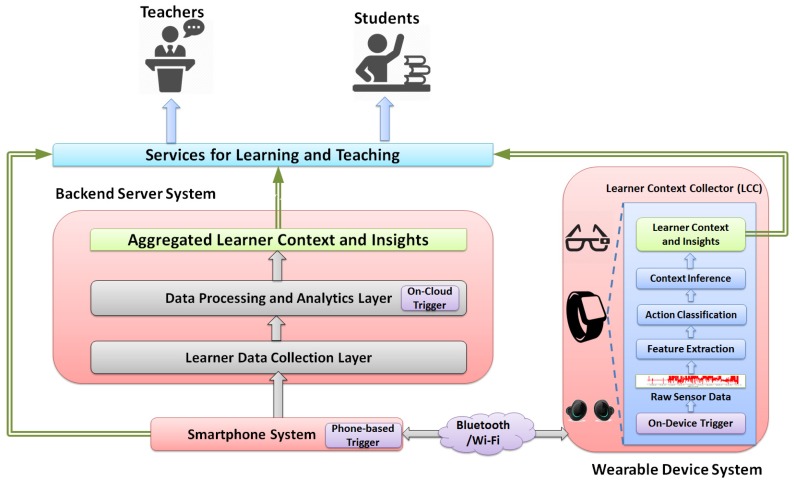
Block diagram of the LEARNSenseframework.

The block diagram of the LEARNSense framework is illustrated in Figure 1, which consists of three subsystems, namely the wearable device system, the backend server system and the smartphone system.

### 3.1. Wearable Device System

As the key component of the proposed framework, this system mainly captures learner’s physical actions and learner context information on different commodity wearable devices, such as wrist-worn and eye-worn devices. More specifically, a lightweight application, called the learner context collector (LCC), is designed for running on these devices: It first gathers the raw data from the built-in sensors on the device, then performs the learner action recognition and, finally, conducts the high-level learner context inference tasks. For example, it first collects the accelerometer data to detect student basic actions in class, such as raising a hand or writing notes, and then infers the student engagement level using the detected actions. The sensor data collection and context inference can be activated and terminated in a non-intrusive way, meaning the entire process does not need the learner’s manual configuration or input.

As mentioned earlier, commodity wearable devices usually have been equipped with multiple built-in sensors. Taking wrist-worn devices as an example, its on-device sensors typically include:

- Accelerometer: measures the linear acceleration in the three axes (namely, *x*, *y*, *z*). Its normal sampling rate ranges from 20 to 100 Hz, and the maximum acceleration range is from 2 to 16 g. In addition, its measurements are usually based on the device coordinates rather than the world coordinates (i.e., Earth reference frame);
- Gyroscope: measures the rotational accelerations along the three dimensions. Its normal sampling rate ranges from 20 to 100 Hz, and the maximum acceleration ranges from 250 to 2000${}^{\circ }$/s;
- Magnetic sensor: measures the direction and strength of the magnetic field, primarily the magnetic field of the Earth. Its normal sampling rate ranges from 20 to 100 Hz;
- Heart rate sensor: measures the heart beating rate using infrared light, and its sampling rate ranges from 0.025 to 1 Hz. For the commodity wrist-worn device, its heart rate sensor detects the amount of light absorbed by the haemoglobin in the blood to detect the volume changes in the blood vessel and then utilizes this to calculate the heart rate.

Besides the above-described four types of sensors, other sensors, such as GPS and temperature sensors, have been embedded in the latest commodity wearable devices and, thus, can provide richer data and information about learners. In addition, when installing the LCC application on the learner’s wearable device, it is necessary to get the learner’s permission for collecting sensor data and performing action recognition tasks.

### 3.2. Backend Server System

This system is the component to aggregate, analyse and store heterogeneous data collected mainly from the wearable device system. It adopts a cloud-based two-layer hierarchical architecture, where each layer has its own unique and independent functionality: (1) The learner data collection layer accepts and manages the incoming learner data from wearable devices, smartphones and other data sources (e.g., from virtual learning environment (VLE)). It provides separate communication interfaces and indexing structures (e.g., spatiotemporally indexed) for receiving and managing a large volume of learner data. The data cleaning and preprocessing are also conducted within this layer. (2) The data processing and analytics layer mainly conducts the data analytics and provides the key analytics results on the aggregate level. Such key results or insights would be directly used to build and support the upper services for learning and teaching. A variety of data mining and sensor data processing techniques can be applied in this layer either in an online manner or offline manner. Besides, this layer also includes a specifically designed module, called the on-cloud trigger, which is used to activate the LCC sensing tasks from the cloud side. The triggering decision is made usually based on the pre-defined conditions, such as the device’s current location.

### 3.3. Smartphone System

This system refers to the learner’s smartphone that has been paired with the wearable device, and it can be used to activate LCC on the wearable device or receive notifications from the backend server system. To conserve the energy of wearable devices, LCC is usually turned off and only activated when the user is at the learning-related locations, such as the classroom or library. Moreover, the smartphone system may also work as a communication gateway between the wearable device system and the backend server system, as some of the existing wearable devices only have short-range communication capability (e.g., only Bluetooth available on the device). However, more and more commodity wearable devices have been equipped with the Wi-Fi or cellular 3G/4G communication interfaces and, thus, can directly communicate with the backend server system without leveraging the smartphone system.

### 3.4. Services for Learning and Teaching

Based on the detected learner physical actions and context information, a variety of upper services can be designed and supplied: different services may require different levels of learner information, from the individual level on a single device to the aggregate level on backend servers, and they may run at different hosts, either on wearable devices or cloud-based clusters (sometimes, maybe even on smartphones). However, their main objective is to improve the learning process, provide effective interventions and optimize the environment for learners.

In short, the above-described three systems under the proposed LEARNSense work cooperatively to accomplish our objectives for learning analytics, which would eventually benefit different stakeholders, including learners, teachers and schools.

## 4. Learner Context Collector Design

As mentioned earlier, we adopt a hierarchical decomposition approach to design and implement a robust application, called the learner context collector (LCC), running on the wearable devices, which aims to accurately and effectively capture learner physical actions and learner context information.

### 4.1. LCC Workflow

The LCC application consists of multiple learner action classifiers, the context inference module and an on-device trigger. The physical action classifiers mainly use different sensor data and the extracted feature sets to identify different types of physical actions. Using the identified learner actions, the context inference module further derives learner context information and key insights. The on-device trigger is used to wake up the LCC application from the sleep status and activate the sensor data collection process.

**Figure 2:**
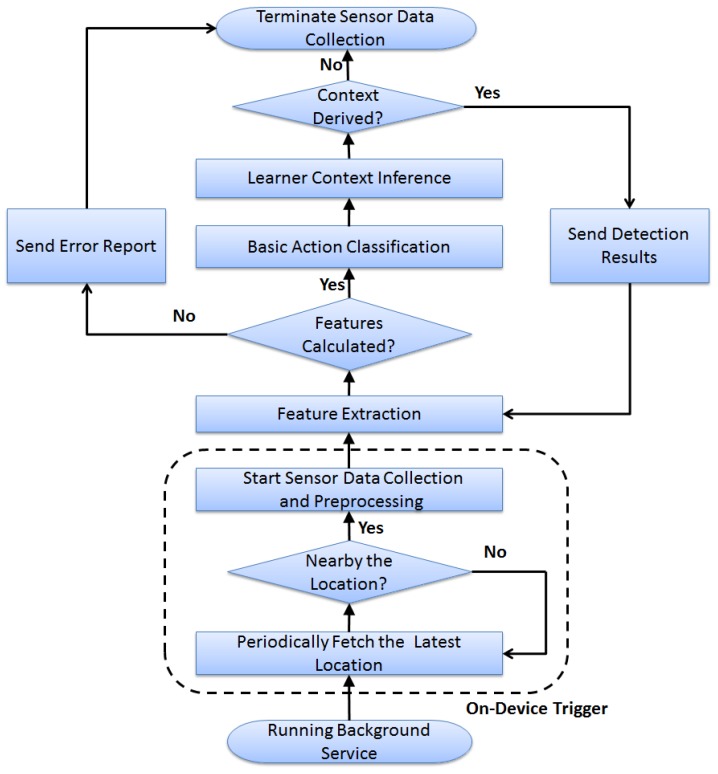
Learner context collector workflow.

As shown in Figure 2, the basic workflow and main components of LCC can be described as below:

1. On-device trigger: The installed LCC registers and runs a background service, which periodically retrieves the current location from the device. If its current location is nearby the pre-defined areas, such as the classroom or library, the trigger would activate the sensor data collection process; otherwise, it keeps LCC sleeping for a specific time period and then checks the latest location again.
2. Data preprocessing: once the sensor data collection is triggered, multiple types of sensors (including accelerometer, gyroscope, magnetic and heart rate sensors) would be activated. LCC usually applies a low-pass filter to the raw measurements and then transforms the readings from the device coordinates to the world coordinates, namely the Earth reference frame.
3. Feature extraction: The transformed sensor data would be used to conduct the feature extraction, where both the time-domain and frequency-domain features are calculated. If the desired features are successfully extracted (meaning all of the calculated feature values fall into their normal ranges), LCC would enable the upper action classification task. Otherwise, LCC would log the error and abort the sensor data collection process.
4. Basic action classification: This may involve one or multiple classifiers to conduct the fine-grained basic action recognition for learners, which mainly uses the supervised learning models, such as decision tree, naive Bayes or artificial neural network. Each classifier may have a number of output options, such as writing or hand-up, and these output options would be used to label each non-overlapping short-duration time window (e.g., 2 s). The sequence of the classifier outputs would serve as the input for the upper context inference module.
5. Learner context inference: This mainly focuses on deriving learner’s high-level physical status or cognitive status using the detected basic actions and other key indicators, such as the learner location or learning time. For example, it may infer that a student is in a good engagement status when consecutive hand-up and stand-up physical actions are detected inside the classroom during the time of a lecture. Compared to the basic action classification, learner context inference may require a relatively long-term time window (e.g., 2 min) to collect enough basic information. The inferred context information can be transferred in a timely manner to external services via the wireless communication interfaces on wearable devices or used directly for the local services running on wearable devices.

In short, our LCC design is generic and can be applied to capture a large number of physical actions and context information from the learner side. Currently, the features and classification models implemented conform to the specific use case and service that we demonstrate, but its development and functionality can be easily extended to other use cases and services.

### 4.2. Data Preprocessing and Feature Extraction

#### 4.2.1. Data Preprocessing

**Figure 3:**
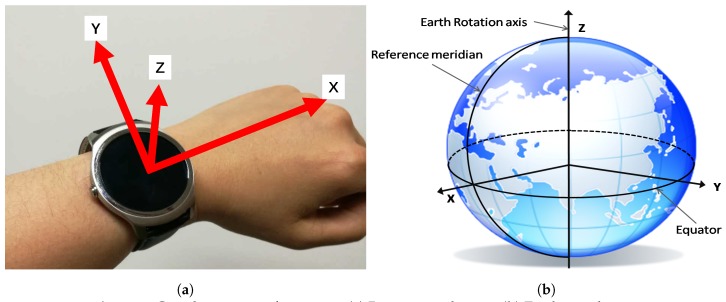
Coordinates transformation: (**a**) Device coordinates; (**b**) Earth coordinates.

As mentioned earlier, the raw sensor readings, especially the accelerometer measurements in the three axes (i.e., *x*, *y*, *z*), are based on the device coordinates, as shown in Figure 3a. Accordingly, the collected accelerometer readings are very much influenced by the device placement and position. To tackle this issue, LCC firstly transforms the raw accelerometer readings from the device coordinates to the Earth coordinates (see Figure 3b) before conducting the feature extraction.

Briefly speaking, the magnetic sensor outputs the magnetic vector in the three axes of the device coordinates in orthogonal directions. This information can be used to obtain the device’s azimuth angle. By fusing the data from the accelerometer and magnetic sensor, it is feasible to detect the orientation of the device. However, this combination has drawbacks like inaccurate readings from the magnetic sensor and noise from the accelerometer. To correct errors caused by the accelerometer and the magnetic sensor, the gyroscope data are used, as it provides rotation speeds relative to the device. Hence, we essentially use three types of sensor to derive the acceleration outputs that are independent of the orientation of a device. The coordinate transformation has been well studied [29,30,31] and can be easily implemented on Android devices using the API provided by Google [32]. Before the sensor data collection and coordinate transformation process, calibration [33] needs to be conducted on each sensor using either the built-in calibration tool from the manufacturer or the third-party ones.

#### 4.2.2. Feature Extraction

After the filtering and coordinate transformation steps, LCC segments the sensor data into non-overlapping fixed-size frames and computes the following features for each frame:

1. Time-domain: a total of 13 commonly-used features for the activity recognition including mean, magnitude of mean, variance, correlation and covariance.
2. Frequency-domain: a total of six commonly-used features for the activity recognition, including spectral energy and entropy.

Table 1 summarizes all of the features implemented and used in the current LCC classifiers. More features can be freely introduced, such as interquartile range and zero-crossings rate in the time-domain, as well as wavelet entropy and wavelet magnitude in the frequency-domain.

### 4.3. Basic Action Classification

To conduct the basic action recognition task, a specific machine learning model needs to be implemented. The model can be selected from the existing supervised learning models, typically including decision tree, support vector machine and naive Bayes. The inputs of the model are selected from the given features during the current time period, and the output of the model would be the classified basic action for this time period.

For different use cases, the LCC may adopt different machine learning models to handle different characteristics of the data. We will elaborate the adopted model and the model training process in the next section, which gives the exemplary use case. The classified basic action would be directly used for the upper learner context inference.

### 4.4. Learner Context Inference

As mentioned earlier, the learner context inference mainly targets deriving learner’s high-level physical or cognitive status using the recognized learner basic actions and other key information, such as the learning location and time. The specifically-designed algorithms or rules usually need be utilized, and the existing context-aware computing techniques can also be introduced to ascertain the learner context information.

Similar to the basic action classification, the learner context inference design usually conforms to the practical use case, and we will elaborate its details in the next section, as well. The inferred learner context information can be either buffered locally on the device or transmitted to the backend server system, which can be used to build a number of upper services.

In short, all of the triggering, preprocessing and feature extraction steps work automatically, and the entire process is transparent to learners. Therefore, the designed LCC is a hassle-free and non-intrusive solution for learner action and context sensing.

### 4.5. Trigger Mechanism

**Figure 4:**
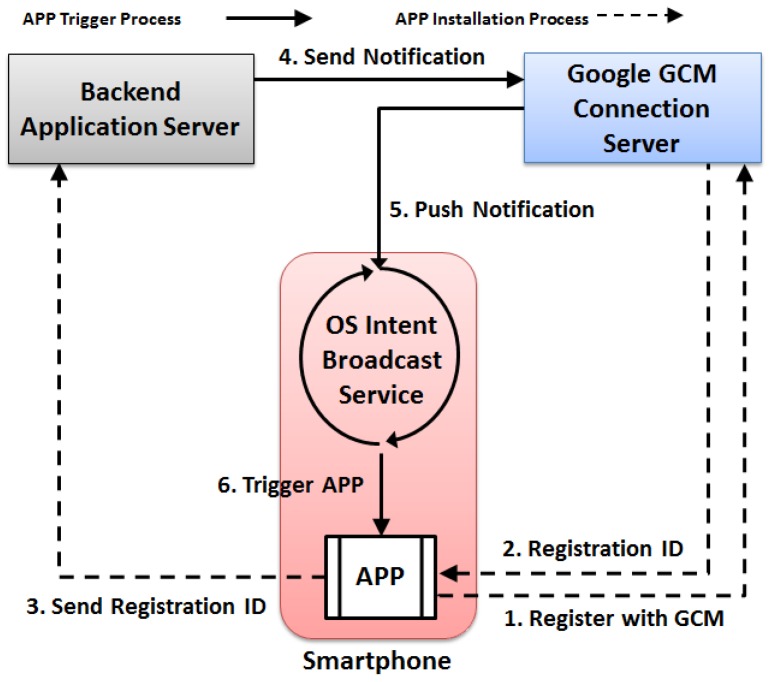
GCM workflow for smartphones.

Besides actively triggered from the wearable device itself, ideally, LCC can also be remotely enabled from the backend server side. Currently, it is still difficult to achieve that due to the limited support from the operating system level. However, it is possible to trigger the paired smartphone remotely from the backend side and then to use the smartphone to enable wearable devices. Such a remote triggering mechanism can be achieved via the so-called Google Cloud Messaging (GCM) service, which now supports both Android and iOS devices. When a GCM message arrives, it can wake up the smartphone from the sleep state and then activate the specific application using the so-called intent broadcast. Figure 4 demonstrates the installation and the triggering process of the current GCM service for smartphones.

When necessary, the backend server system would send out the GCM message carrying the classroom location to enable the paired smartphones. The enabled smartphones then push the notifications to their paired wearable devices. The wearable devices would then firstly run a background process to retrieve the current device location. If the device’s current location is close enough to the classroom location, LCC would then initiate the sensing and analytics pipeline. Otherwise, LCC acknowledges its current location to the smartphone and continues to sleep, which actively saves the energy consumption on the wearable device side.

In short, all of the triggering, preprocessing and feature extraction steps work automatically, and the entire process is transparent to learners. Therefore, the designed LCC is a hassle-free and non-intrusive solution for learner action and context sensing.

## 5. Exemplary Use Case

The proposed LEARNSense framework and its LCC can be applied to a number of use cases and, accordingly, build the services for learners and instructors. To demonstrate their design, operation and performance, we present an exemplary use case called the class engagement analyser.

### 5.1. Problem Description

Collecting student engagement status in class at the individual level is critical and meaningful, as such learner context information would not only help to monitor and understand the learning pattern and process on distinct students, but also help teachers to conduct interventions and evaluations. Obviously, it is almost infeasible for teachers to accurately and concurrently collect the engagement status of every student in a manual way. Using a vision-based system with a video camera possibly provides an alternative solution, but it requires building a fixed infrastructure with enough cameras. Moreover, the vision-based system has a number of other issues, such as a low performance and accuracy in natural lighting conditions [24].

Our LEARNSense framework and its LCC provide a natural and effective solution to tackle this problem. In this section, we present the use case with a special focus on how the implemented LCC detects student physical actions in class and then infers their engagement status. Besides, the wearable device essentially supplies a natural platform to build the upper service, such as a private intervention to specific student, and we will demonstrate it in this section, as well.

### 5.2. Wearable Device System Implementation

#### 5.2.1. Hardware Selection

Among multiple types of commodity wearable devices available on the market, we choose the wrist-worn device for this use case, as the wrist-related physical actions are crucial to understand the student behaviour and engagement in class. In practice, we adopt one type of wrist-worn device, the smartwatch called Ticwatch [34], which is equipped with the desired sensors, including accelerometer, gyroscope, magnetic and heart rate sensors. The sampling rate for accelerometer, gyroscope and magnetic sensors is set to 50 Hz and for the heart rate sensor is set to 1 Hz. The maximum acceleration ranges from −4 to 4 g, and the maximum gyroscope acceleration ranges from −720 to 720${}^{\circ }$/s. Moreover, Ticwatch also comes with GPS and cellular 3G/4G modules, which greatly facilitate locating the device and communicating with the backend server system without a paired smartphone. It is running the Android operating system with open SDK [35] for developers and can be easily purchased from the market at the price of less than 200 U.S. dollars each.

#### 5.2.2. Basic Action Classification

Running at the root of the LCC hierarchy, the current implementation of LCC mainly detects four typical student physical actions as follows:

1. Writing: hand keeps moving on a horizontal surface, which is used to detect a student taking down notes in class;
2. Hand-up-down: hand is raising up or being put down, which is used to detect a student notifying the teacher to ask or answer questions;
3. Other-moving: hand keeps changing its positions on both the vertical and horizontal planes, which is used to detect the student’s other physical actions (excluding the above ones);
4. Stationary: hand is relatively stable and fixed in one place, which is used to detect a student in a motionless status.

**Figure 5:**
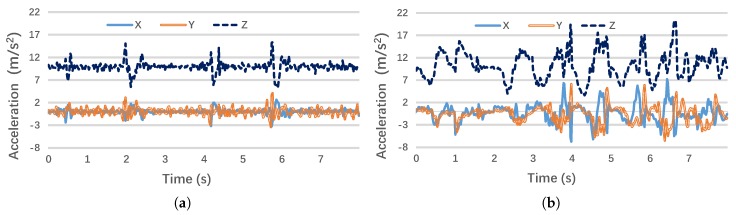
Accelerometer data from different actions: (**a**) Writing actions; (**b**) Hand-up-down actions.

The basic action classifier mainly employs the accelerometer data, which have been transformed to the Earth coordinates using the gyroscope and magnetic sensor measurements. Figure 5a,b illustrates the transformed three-axis accelerometer outputs when a student conducts writing and hand-up actions, respectively: compared to the first writing action, the hand-up-down action shows much higher variances on both the horizontal plane (*x*-axis and *y*-axis) and the vertical plane (*z*-axis). Therefore, their variance in time domain and spectral energy in the frequency domain may serve as the most effective features to differentiate these two basic actions.

To classify the above four actions, the basic action classifier utilizes all of the time-domain and frequency-domain features describe in Table 1, and thus, its feature space consists of 19 features. We then collect the training data (which will be elaborated in the following evaluation subsection) to build the classifier: we evaluated several commonly-used supervised learning models, such as decision tree, naive Bayes and support vector machine, and we adopt the decision tree C4.5 for the current classifier. This is mainly because the resulting tree model can be easily interpreted and implemented on wearable devices.

The built classifier periodically outputs one physical action to label the current time window. The time window size for this classifier needs to be relatively small, as a large time window may cover multiple student basic actions and accordingly decrease the classification accuracy. We will show the detailed classifier accuracy in the following evaluation part. Finally, the sequence of these student basic actions can be sent to the upper context inference module to further infer student engagement status and learner context information.

#### 5.2.3. Learner Context Inference Module

With the objective of inferring the learner’s physical or cognitive status related to the learning process, the context inference module in this use case mainly focuses on differentiating the student learning states in the class. Besides the detected four basic actions from the built classifiers, the wrist-worn device also provides another form of physical action from the student side, namely the heart beating rate from the heart rate sensor. By leveraging all of these physical actions’ information, we adopt the heuristic approach to design a practical algorithm, called the student state distinction (SSD) algorithm, to infer the three distinct states that may reflect the distinct student engagement levels in class.

The basic idea of the SSD algorithm is simple: given any time period, if the specific basic actions, especially the hand-up, hand-down and writing actions, are frequently detected, and meanwhile a high variance of the heart beating rate is observed, the corresponding student and his or her current state can be distinguished from others. As the inputs of the SSD algorithm, $N_{w}$, $N_{h}$, $N_{m}$ and $N_{s}$ are the number of the detected writing, hand-up-down, other-moving and stationary actions during the time period, respectively, and $\Phi_{h}$ is the set of heart rate measurements during the time period. The complete algorithm is shown in Algorithm 1.
**Algorithm 1** Student state distinction (SSD) algorithm.**Input:**
$N_{w}$, $N_{h}$, $N_{m}$, $N_{s}$, $\Phi_{h}$, time period length *T*, threshold $\theta_{n}$ and $\theta_{h}$, weights $w_{w}$, $w_{h}$, $w_{m}$, $w_{s}$.**Output:** Inferred learner state for current time period. 1: ${\bar{N}}_{T}=\frac{w_{w}N_{w}+w_{h}N_{h}+w_{m}N_{m}+w_{s}N_{s}}{T}$; 2: $\sigma_{T}=std\left(\Phi_{h}\right)$; 3: **if**
$\bar{N}>\theta_{n}$ and $\sigma_{T}>\theta_{h}$
**then** 4:   Infer *learner active state* during current time period; 5: **else if**
$\bar{N}<\theta_{n}$ and $\sigma_{T}<\theta_{h}$
**then** 6:   Infer *learner inactive state* during current time period; 7: **else** 8:   Infer *unclear state* during current time period;

The given SSD algorithm mainly consists of three steps: Firstly, it calculates the weighted arithmetic mean over the number of different actions. $w_{w}$, $w_{h}$, $w_{m}$ and $w_{s}$ are the predetermined weights for each action, and we will elaborate them in the evaluation part. After that, the value is divided by the given time period length *T*, and we call the computed value ${\bar{N}}_{T}$ as the student action score. Secondly, SSD calculates the standard deviation of the heart rate measurements $\Phi_{h}$ during the time period, which quantifies the fluctuation of the student heart beating. The high variance of the heart rate intuitively reflects the student exciting status. Note that we do not use the absolute heart rate values as the criteria, as they show a larger difference even from two students in the same status. Finally, the SSD algorithm infers the so-called learner active state (LAS), when both ${\bar{N}}_{T}$ and $\sigma_{T}$ are bigger than the threshold values $\theta_{n}$ and $\theta_{h}$, respectively. On the other hand, if both values are smaller than the threshold values, the SSD infers the so-called learner inactive state (LIS) for the current time period. For any other situations, the SSD algorithm simply infers them as the unclear state (US). To store and batch process the detected student basic actions and heart rate deviation, we buffer all of the received data during the time period *T*, which is set as a fixed-size moving time window. The determination of the time window size and the threshold values used in the SSD algorithm will be elaborated in the evaluation part.

In short, the learner context inference module jointly considers the student physical actions together with student heart rate information to categorize students into three states, which reflect their engagement status in class. The entire module has been implemented on the wearable device and can be easily extended to the backend server side.

### 5.3. Backend Server System Implementation

For all of the smartwatches with the running LCC, the inferred student states would be transferred back to the backend server system in real time. As the Ticwatch hardware has been equipped with a built-in cellular module, it is feasible to use 3G or 4G service to send the messages without the help of the smartphone system. Each message consists of a four-tuple payload $\langle id,desc,time,loc\rangle$, where id can be the student ID, desc is the student state, namely LAS, LIS or US, time is the current timestamp and loc is the device current location.

To handle the incoming messages from multiple smartwatches and conduct the analytics on the aggregated information, we specifically design an algorithm, called the class engagement analysis (CEA) algorithm. Briefly speaking, the CEA algorithm works in a message-driven way, meaning each newly-arrived LCC message would invoke the algorithm once. The algorithm constructs a hash table $H_{T}$, whose key is the student ID (i.e., *id*), and its value includes a so-called engagement ratio, denoted as *r*. The ratio *r* is defined as the student LAS duration to the LIS duration, and thus, a bigger ratio value indicates a higher engagement level in class. The CEA algorithm mainly runs on the server side, and the complete algorithm is shown in Algorithm 2.
**Algorithm 2** Class engagement analysis (CEA) algorithm.**Input:** A new LCC message $\langle id,desc,time,loc\rangle$, hash table $H_{T}$, location *q*, time length *T*, small positive number ε.**Output:** Hash table $H_{T}$ with the engagement ratio *r*. **if**
id is not a key in $H_{T}$
**then**  **if**
loc∈q AND desc = *LAS*
**then**   $T_{LAS}\leftarrow T$; $T_{LIS}\leftarrow \varepsilon$;  **else if**
loc∈q AND desc = *LIS*
**then**   $T_{LAS}\leftarrow 0$; $T_{LIS}\leftarrow T$;  $r\leftarrow \frac{T_{LAS}}{T_{LIS}}$;  Insert a new key-value pair $\left(id,\;\left[q,r,T_{LIS}\right]\right)$ into $H_{T}$; **else if**
id is an existing key in $H_{T}$
**then**  Get the value $\left[q,r,T_{LIS}\right]$ from $H_{T}$ using id;  **if**
loc∈q AND desc=LAS
**then**   $r\leftarrow \frac{r*T_{LIS}+T}{T_{LIS}}$;  **else if**
loc∈q AND desc=LIS
**then**   $r\leftarrow \frac{r*T_{LIS}}{T_{LIS}+T}$; $T_{LIS}\leftarrow T_{LIS}+T$  Update the latest key-value pair $\left(id,\;\left[q,r,T_{LIS}\right]\right)$ into $H_{T}$;

As mentioned earlier, the given CEA algorithm works in a message-driven way. Whenever an LCC message is sent from a new smartwatch, i.e., id is not an existing key in $H_{T}$, the CEA algorithm would then check whether the device location loc is inside or nearby the given location *q* (typically a classroom) and whether the message comes with the LAS description. If both conditions are true, the algorithm would use the time length *T* (used in the SSD algorithm) and a small positive number ε to calculate the engagement ratio *r*. After that, the algorithm inserts a new key-value pair, i.e., $\left(id,\;\left[q,r,T_{LIS}\right]\right)$, into the hash table $H_{T}$. Similar logic applies to the LCC message with the LIS description. Whenever id is an existing key in $H_{T}$, meaning the LCC message sent from a smartwatch already reported before, the algorithm would directly retrieve the stored values from $H_{T}$, i.e., the location *q*, engagement ratio *r* and LIS duration $T_{LIS}$. It then checks whether the device is still at the same location and the delivered message with the LAS or LIS description. If both conditions are true, the algorithm would re-calculate the engagement ratio and update it in the hash table $H_{T}$ accordingly. Note that the current CEA algorithm purposely discards the messages with the US description, as the unclear state is not significant to evaluate student engagement level in class.

In the practical implementation, all of the corresponding variables used in the CEA algorithm would be periodically re-initialized. Moreover, the indoor localization techniques [36], such as the RFID-based method, can be introduced to determine the device location with better accuracy.

### 5.4. Services for Learning and Teaching

Computed by the CEA algorithm, the engagement ratio *r* and the LIS duration $T_{LIS}$ can be used to build and support a variety of upper services. For example, when the consecutive LIS states are detected and the duration $T_{LIS}$ is long enough, this indicates that the corresponding student is highly possibly in a low engagement status. In that case, the system can provide services to deliver proper interventions to the student. On the other hand, when the computed ratio *r* is found always high during the class time, this indicates the corresponding student has a high engagement status for that class. The system can thus provide services to incentivize and encourage the student after class.

**Figure 6:**
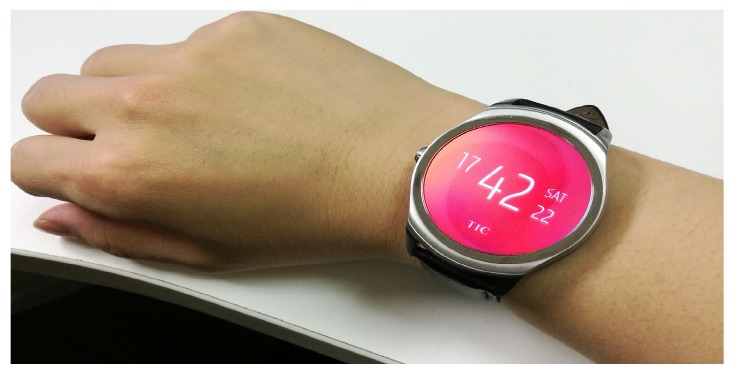
Smartwatch vibrating in classroom

**Figure 7:**
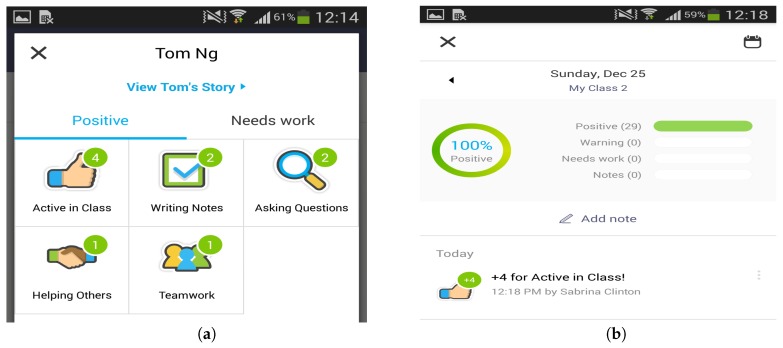
Incentive mechanism of the class engagement analysis (CEA) service. (**a**) Sample report for the teacher; (**b**) Sample report for the student.

The current system mainly implemented the two services as described below:

1. Online intervention: When consecutive LIS states are detected from the same student (currently, the threshold is set to 10 min), the system would automatically trigger the vibration function on his or her smartwatch. It is an explicit signal to remind the student, but in a private way. Compared to the teacher directly warning the student in class, this service would help to avoid the embarrassment of students and prevent awkward moments. Figure 6 shows that the smartwatch is vibrating with red colour on the screen to remind the student with a low engagement ratio.
2. Offline incentivizing: When a student always receives a high engagement ratio *r* (currently, the threshold is set to 3.0) for the same class, the system would provide the incentives on his or her personal profile by leveraging on ClassDojo [37]. ClassDojo is a widely-used and free classroom behaviour management tool that allows instructors to supply feedback to students regarding both individual and group behaviours. The feedback on ClassDojo can be edited by teachers and easily shared with students and their parents. As shown in Figure 7, the incentivizing points have been added to the student profile on ClassDojo when a high engagement ratio is detected, and then, the service generates the reports for the teacher (see Figure 7a) and the student (see Figure 7b).

Besides the service lists above, a variety of other services can be designed and implemented based on the derive student state and physical action information in class. Moreover, the threshold values used in the these two services can be easily adjustable by teachers according to their own teaching strategy.

### 5.5. System Evaluation

We conduct a comprehensive evaluation on the implemented system and services, where we adopt both qualitative and quantitative methods to account for their performance.

#### 5.5.1. LCC Evaluation

As mentioned earlier, we implemented the Android version of the designed LCC and tested it on the Ticwatch hardware, which runs the Android 5.1 operating system. The sampling frequency for accelerometer, gyroscope and magnetic sensors are set to 50 Hz, and the heart rate sampling frequency is set to 1 Hz.

To build and train the model for recognizing the four basic actions in class, the first step is to collect enough training data. In practice, the training data are collected by multiple participants at three different schools under the following natural scenarios:

- The participants quietly listen to a lecture or are napping with a comfortable posture in class. The data are collected by 18 participants during a two-week period, and all of the participants wear a smartwatch with the running LCC.
- The participants freely conduct other activities in class, such as grouping together for discussion or making presentations. The data are collected by 16 participants during a three-week period, and all of the participants wear a smartwatch with the running LCC.
- The participants keep writing down notes in class. These data are collected by 17 participants during a two-week period, and all of the participants wear a smartwatch with the running LCC on their writing hand.
- The participants raise their hands to ask or answer questions in class. The data are collected by 14 participants during a two-week period, and all of the participants wear a smartwatch with the running LCC on their raising hand.

All of the participants are required to record the ground truth, and we allow the participants simply to record their negative activities as other actions. Moreover, for collecting the ground truth of the short actions, such as hand-up and hand-down, we assign one independent observer to help the participants to record the ground truth. In addition, for some of the ground truths having the matched video data, we also use the video information to validate and find that the corresponding ground truth data are reliable and accurate. The sex ratio is controlled to approximately 1:1 to ensure that the results are not sensitive to gender. Using the collected training data, we build the basic action classifier with the decision tree model, and Table 2 gives the evaluation results: we see that the F1 scores of all actions are around 0.9, indicating that the classifier can well distinguish the four types of basic actions, although the F1 score of hand-up-down is slightly lower. The overall classification accuracy is 91.5% with 10-fold cross-validation, and such a high accuracy ensures the feasibility and quality to further infer the student states using these detected physical actions. Table 3 gives the corresponding confusion matrix.

By using the trial-and-error method, we finally set the time window size for this classifier to 1000 milliseconds, i.e., 1 s, as a larger time window may decrease the classification accuracy due to multiple basic actions being included, and a smaller time window may not be long enough to include a single basic action.

Table 4 further gives the classification performance using the three different models. Compared to naive Bayes and support vector machine models, the decision tree model always achieves a higher F1 score, although the naive Bayes has a slightly higher F1 score for the stationary action.

#### 5.5.2. Context Inference Evaluation

To determine the weight values used in the current SSD algorithm, we asked the experienced teachers to select the typical active and inactive students from their classes, where eight active and eight inactive students were chosen. We manually counted the time duration of each action conducted by active students and inactive students respectively using the recorded video. After that, we computed the ratio of the average duration of active students to inactive students for each action and used the normalized ratios to set the weight values in the SSD algorithm. Based on the above-described experiments and computations, we finally assign 12.5, 9.0, 6.8 and 1 to the weights $w_{w}$, $w_{h}$, $w_{m}$ and $w_{s}$, namely the writing, hand-up-down, other moving and stationary, respectively. Generally speaking, we assign the highest weight value to writing action and the lowest weight value to stationary, as different actions show different capabilities to infer student active status. The assigned weight values are used to compute the student action score in the SSD algorithm, which are adjustable according to different use cases and scenarios. Since each student may have his or her own activity and heart beating patterns, the two threshold values in the SSD algorithm, namely $\theta_{h}$ and $\theta_{n}$, can be set as the personalized parameters: for each individual student, we can compute his or her 25th percentile values of the heart beating standard deviation and student action score in the last seven days and use these two values as the thresholds. In the current implementation, we simply set these two thresholds using the 25th percentile of all of the collected student data in the experiment. In addition, the time window length *T* for the SSD algorithm needs to be relatively large, as it should cover multiple student basic actions. In our practical implementation, we set its length to 5 min, meaning it covers 300 detected basic actions in a row. To validate the designed SSD algorithm and its parameters, we selected another eight students and collected their data using both the LCC and the recorded video. We asked the experienced teachers to manually label the three learner states (i.e., active, inactive and unclear) via the observation of the video episodes: each video episode lasts around 5 min, and in total, 127 episodes are labelled as the ground truth. The validation results show that 109 episodes were correctly classified by the current SSD algorithm, meaning the overall accuracy achieves 85.8%.

After the parameter determination and the algorithm validation, we conducted another experiment for the context inference module. The experiment in total involves 24 participants (11 female and 13 male), whose age ranges from 11 to 13 years old. The participants are manually divided into two groups according to the teacher’s observation on their daily class performance: Group A is the normal students, and Group B is active students who usually have a higher level of engagement in class. The experiment was conducted during a normal lecture-based class, involving a number of question-answer interactions between the teacher and students.

**Figure 8:**
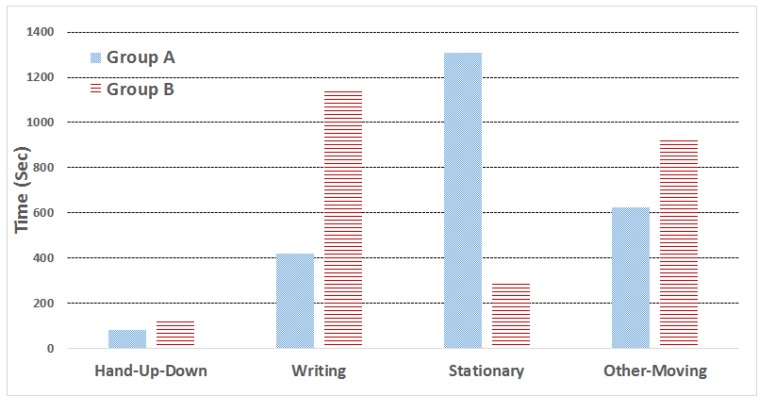
Average duration of the four basic actions.

Both groups were wearing the smartwatches with LCC during the class time, and Figure 8 shows the average duration of the detected four basic actions in terms of different groups in one class (around 40 min, i.e., 2400 s). We see that Group B has a much longer duration of writing and other moving actions than Group A, and meanwhile, the average stationary duration of Group A is significantly higher than Group B. Such results, to a certain degree, illustrate that these three actions can serve as good indicators to infer the student activity levels. Furthermore, it is a little bit surprising that for the hand-up-down action, Group B only exhibits a slightly longer duration than Group A (approximately 80 s for Group A and 120 s for Group B), which may be caused by the lecture settings (not so many questions and interactions in class), as well as the classification errors of LCC.

**Figure 9:**
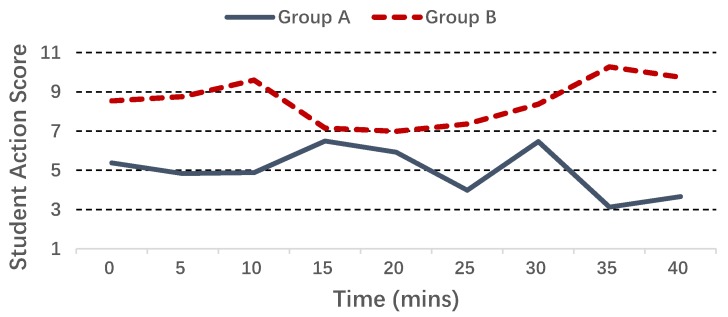
Average student action score of two groups.

**Figure 10:**
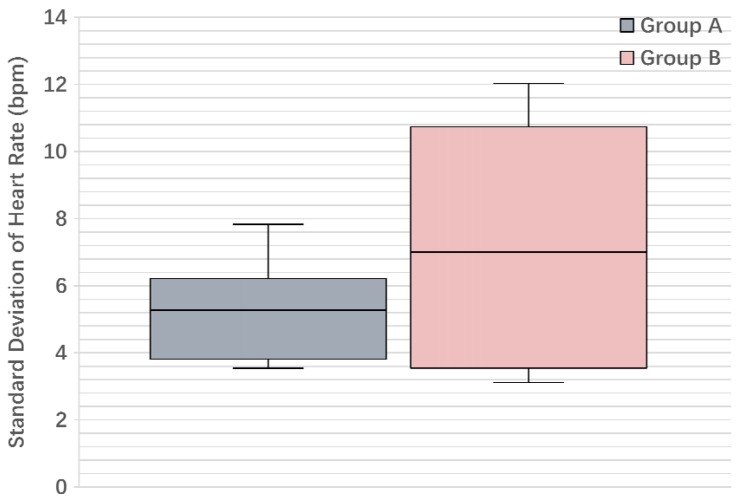
Boxplot for the standard deviation of heart rate.

We run the SSD algorithm on the two groups of students, and Figure 9 shows the average student action score ${\bar{N}}_{T}$ during one class time: we see that clearly, the average score of Group B is always higher than Group A, although the two scores get close to each other in the middle of the class. We also conducted the two-tailed *t*-test [38] on the two groups of student action scores: the *p*-value for their means is 0.032, and Table 5 gives other key information of the student action scores, including the F statistic and the degrees of freedom. The above results verify that the two groups are significantly different from each other on the defined student action score. Furthermore, Figure 10 shows the boxplot for the standard deviation of heart rate: we see that Group B has a higher median value than Group A (even higher than the upper quartile of Group A), which partially explains why we introduce the heart rate deviation in the SSD algorithm.

#### 5.5.3. Service Evaluation

**Figure 11:**
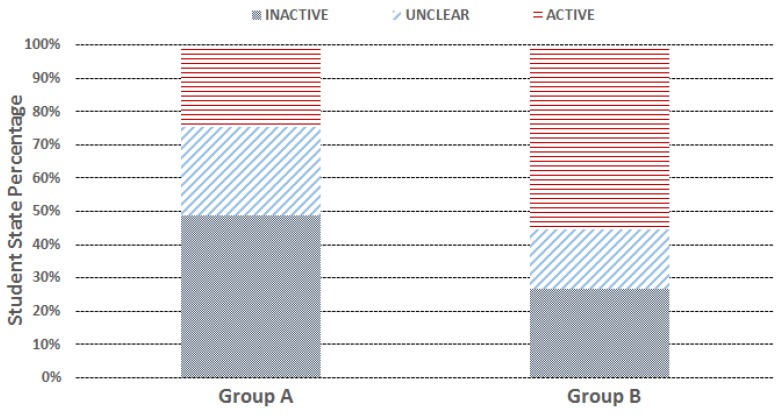
Distribution of student state duration

We run the CEA algorithm on both groups for the built services, and Figure 11 shows the distribution of the aggregated student state duration: we see that Group B’s LAS duration is much higher than Group A, and meanwhile, its LIS duration is significantly lower than Group A. In addition, a certain percentage of the durations (around 25% for Group A and 20% for Group B) are inferred as the US durations. Using the above-derived LAS and LIS information, the built services provide: (1) the interventions through vibrating function on the smartwatches to remind the inactive students; and (2) the offline incentivizing through adding points on the student ClassDojo profiles to encourage the active students. Note that a certain time delay exists for the intervention service, which is mainly due to the SSD algorithm needing to buffer the student action information for a period of time (currently set to 300 s), and then determine the current student state. This time delay is unavoidable, as the system needs to accumulate enough action information to determine the student state, but the buffering duration can be further reduced when necessary.

We further conducted the evaluation by the survey on the participating students. The evaluation questions are designed partially based on the national survey of student engagement [39], where the questionnaire items use a five-point Likert scale, i.e., from 4 = strongly agree to 0 = strongly disagree), and reports the mean (MN) and standard deviation (SD). We collected 24 questionnaires to gather feedback on the use of our service and the perceived usefulness and impact on their learning. Table 6 provides some key responses split by student gender, which mainly shows that the respondents agree on the positive impacts on their learning and that the data collection process does not interfere with the learning process. Moreover, the respondents were overall satisfied with the use of the system and service (MN = 3.7, SD = 0.55). We monitored all of the sessions of the experiments, and the above results are in line with our observations.

Lastly, we also conducted the survey on the teacher side, and in total, five questions are included using the same five-point Likert scale. We collected 13 questionnaires from the teacher side, and the respondents overall felt the service is helpful for identifying the student physical actions (MN = 3.54, SD = 0.51) and their engagement status on the individual level (MN = 3.6, SD = 0.65). Moreover, we conducted a face-to-face interview with the 13 teachers, and all of them agree that the built system does not introduce any additional burden on their side, except managing the ClassDojo profiles for the students.

## 6. Discussion

### 6.1. Understanding Learner and Learner State

In our exemplary use case, using the physical actions to derive the learner states and the class engagement is mainly on the basis of the heuristic approach. It may need further support and explanations from the theoretical perspective. Cognitive psychology and human computer interaction (HCI) fields offer a number of developed frameworks and models that are possibly introduced into our LEARNSense framework. For example, human information processing (HIP) [40] supplies the methods to conceptualize how the human mind works: the basic idea is that human behaviour is a function of several ordered processing stages, meaning it is like a system that can be analysed in terms of subsystems and their interrelation. A widely-accepted and well-known model, called the model human processor (MHP) [41], may help to understand why learner’s physical actions can help to infer human cognitive status, such as the class engagement status.

**Figure 12:**
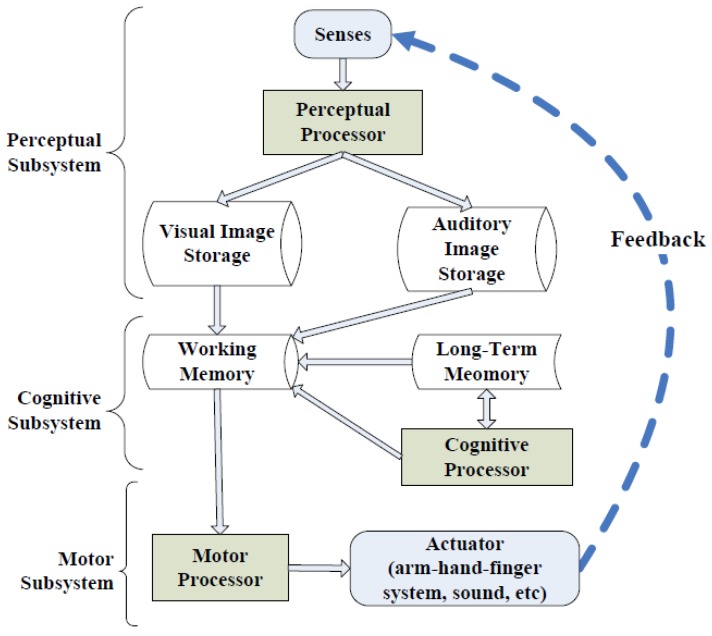
Framework of the model human processor (MHP).

As shown in Figure 12, MHP consists of three interacting subsystems: perceptual subsystem, cognitive subsystem and motor subsystem. Each subsystem has its own processors and memories. The perceptual subsystem is equipped with sensors and associated buffer memories for collecting and temporarily storing external information. The cognitive subsystem accepts symbolically-coded information from the memories of the perceptual subsystem and then decides on how to respond. Finally, the motor subsystem carries out the response and takes action. The MHP models the information processing of humans as a sequential or parallel operation of these three MHP subsystems. Furthermore, MHP provides two principles: one principle, called rationality principle, meaning that the human’s behaviour is based on rational activity and will not randomly and arbitrarily change from one state to another; the other principle, called the problem space principle, indicates that all rational activities serve to achieve the human’s goals and tasks, bounded by the human’s knowledge and processing ability.

For the human cognitive subsystem, it is still difficult to accurately define and differentiate its status, even using electroencephalography (EEG) [42] to record the brain’s spontaneous electrical activity. However, MHP demonstrates that the motor subsystem follows the recognize-act cycle of the cognitive processor, which means monitoring the motor subsystem can directly help to estimate the status of the cognitive subsystem. In other words, monitoring a human’s physical actions may not only help to detect the working status of the motor subsystem, but also may help to infer that the corresponding cognitive subsystem is working. Moreover, MHP considers the arm-hand-finger system as the most important actuator in the motor subsystem, which may explain why wrist-worn devices can be a proper tool of capturing learner’s physical actions under the LEARNSense framework.

Moreover, for the perceptual subsystem, MHP shows that the most important memories for the human perceptual processors are the visual image storage and the auditory image storage. Therefore, the commodity wearable devices for eyes and ears, such as Google glass [4] and Bragi headphones [43], may serve as another significant clue to infer the working status of learner’s cognitive subsystem.

In short, the cognitive psychology models like MHP, to a certain extent, explain and validate our LEARNSense framework design, especially using the wearable devices to recognize learner physical actions and then use them to further infer learner context including both physical and cognitive status.

### 6.2. Current Limitations

While the MHP model reveals the strong connections between human actions and cognitive status, it does not mean the derived learner states, including both LAS and LIS, can be simply interpreted as the active or inactive status on the cognitive level. In other words, the derived LAS and LIS essentially depict the learner’s physical engagement status in class and cannot adequately reflect his or her cognitive engagement status, where cognitive engagement emphasizes the mental efforts of learners more. As a result, our current LCC may not be able to capture some engagement cases: for example, a student concentrating on the class, but motionless and with a low heart rate variance. For these cases, more types of wearable devices and sensors can be considered, such as the eye-worn devices like Google glass for eye tracking, to recognize more types of physical actions, including both explicit and implicit actions. Accordingly, more advanced sensor fusion techniques and data mining models might need to be adopted in the LCC design.

On the other hand, in our current LCC design, it transforms the accelerometer data from the device’s coordinates to the world coordinates, and thus, the physical placement of the smartwatch device does not greatly influence the physical action classification results. However, the smartwatch is required to be worn on the specific hand when detecting the hand-up-down and writing actions. In addition, similar to the most accelerometer-based activity recognition systems, our classification techniques exhibit poor performance when the device is subject to usage-induced artefacts, such as the student casually swinging or frequently rotating the hand. The most common approach is to simply discard the sensor data when such artefacts are detected. Currently, we simply use the non-overlapping time window to classify the student actions, and the time window with a proper ratio can be implemented to further improve the accuracy of the classification model. Moreover, the data collection process does not strictly include independent observers to record the ground truth, which may introduce biased data and affect the model accuracy. To build the next version of LCC and its student action classification models, at least two independent observers are needed to record the ground truth during the entire data collection process.

For the SSD algorithm design, the recent studies [44,45] from learning science show that different overt learning actions reflect different stages of the human cognitive process, and thus, they partially support the current design of the SSD algorithm. However, the current design of the SSD algorithm and its validation still can be further improved in future work, such as using the machine learning algorithms to build a more accurate model for student state classification (e.g., logistic regression or tree-based models) and using K-fold or leave-one-out cross-validation to evaluate rather than the current holdout method.

In addition, some of the empirical results presented in this work are based on the offline analytics, using traces of the collected data, and replaying them through the developed LEARNSense modules. While such offline analytics is adequate for our goals (of demonstrating the proposed framework and exemplary use case), additional modest system enhancements may be needed to support a more real-time implementation. For example, the incentivizing points for the student ClassDojo profile, computed by the backend server system, are currently added in a manual way by teachers. Automating this process by enabling the communication between our backend server and the ClassDojo service can be one of the interesting implementation issues for future work.

### 6.3. Future Extensions

While our framework and exemplary use case can be explained by the above-described MHP model in a general way, how to better connect the established cognitive models with the detected fine-grained learner actions is still an open and interesting research problem. For example, the implemented SSD algorithm in the use case simply employs the two fixed threshold values to differentiate learner states, which can be refined when the cognitive models and parameters are properly applied. We believe that a deeper understanding of the learners and their cognitive mechanism would directly help to explain their physical actions and derive their context information, in turn establishing a way to impact and optimize their learning process.

The proposed LEARNSense framework is currently only looking at the physical actions and context information from the learner side, while information from the teacher side would be also significant and valuable. For example, the teacher’s physical gestures and interaction activities in class, such as explanation or questioning, may directly influence the student learning status and effect. Using the commodity wearable devices to capture and aggregate the information from both sides of teachers and students may pave a new way toward building learning models and eventually enabling novel systems and services for learning analytics.

In addition, as the hardware cost (including sensors and processors) keeps dropping down, the price of commodity wearable devices can be expected to reach a lower price, which would be affordable for most of the student’s families. Considering other attractive functionalities of commodity wearable devices (such as locating young students and tracking their fitness), we believe it would enable teachers and parents to practically deploy the proposed LEARNSense framework in a large-scale educational environment.

## 7. Conclusions

We have introduced the LEARNSense framework, which aims to provide learning-related context information and services by harnessing the commodity wearable devices. We designed and implemented modules for identifying the learner basic actions and inferring learner context on wearable devices and the backend server system. The built-in sensors on wearable devices, typically including accelerometer and heart rate sensors, together with the data mining techniques are employed to build the LCC application. We demonstrated the practical embodiments of the framework and the promise of the LCC via an exemplary use case: the class engagement analyser is able to accurately recognize the basic student actions in class and then infer the learner context, i.e., learner active state and learner inactive state, to describe the engagement level in class. With the inferred learner context information, we further design the services that provide proper interventions and incentivizing feedback to students. The comprehensive evaluation results confirm that our original design goals are achieved.

On a broader canvas, the LEARNSense framework reveals the feasibility and significance of employing the commodity wearable devices and learner physical actions for learning analytics. We believe that more use cases and services can be developed using this framework or a similar design philosophy.

## Figures and Tables

**Table 1:** Implemented features on the learner context collector (LCC).

	Feature Name	Number
**Time Domain**	Mean: $\bar{x}$,$\bar{y}$,$\bar{z}$	3
	Magnitude of Mean: $\sqrt{{\bar{x}}^{2}+{\bar{y}}^{2}+{\bar{z}}^{2}}$	1
	Variance: var(x),var(y),var(z)	3
	Correlation: corr(x,y),corr(y,z),corr(x,z)	3
	Covariance: cov(x,y),cov(y,z),cov(x,z)	3
**Frequency Domain**	Energy: $\frac{\sum_{j=1}^{N}\left({m_{j}}^{2}\right)}{N}$ computed for *x*, *y* and *z* respectively, where $m_{j}$ is FFT component.	3
	Energy: $-\sum_{j=1}^{n}\left(p_{j}*\log\left(p_{j}\right)\right)$ computed for *x*, *y* and *z* respectively, where $p_{j}$ is FFT histogram.	3

**Table 2:** Accuracy of the basic action classifier.

	Precision (%)	Recall (%)	F1 Score
Writing	87.3	93.2	0.902
Hand-Up-Down	85.7	93.8	0.896
Other-Moving	93.8	88.2	0.909
Stationary	97.6	95.3	0.965

**Table 3:** Confusion matrix for action recognition.

		Predicted Class			
		Hand-Up-Down	Stationary	Writing	Other-Moving
**Actual Class**	Hand-Up-Down	630	1	20	21
	Stationary	22	862	0	20
	Writing	0	1	1156	84
	Other-Moving	83	20	148	1890

**Table 4:** F1 scores for different classification models.

	Hand-Up-Down	Stationary	Writing	Other-Moving
Decision Tree	0.896	0.965	0.902	0.909
Naive Bayes	0.890	0.970	0.894	0.908
Support Vector Machine	0.865	0.940	0.885	0.902

**Table 5:** Key statistics of the student action score.

	Group A	Group B
Mean	5.775	8.45575
Variance	2.958409	1.457806
Degree of Freedom	11	11
F statistic	2.029357	
F critical (alpha = 0.05)	2.81793	

**Table 6:** Some key responses split by student gender.

Question	All (n = 24)	Female (n = 11)	Male (n = 13)
I feel better when teacher reminds me by letting the smartwatch vibrating on my wrist than speaks out my name in class.	3.75 (SD = 0.44)	3.818 (SD = 0.40)	3.692 (SD = 0.48)
I feel more confident and happy on learning that subject, when I receive online points because of my activeness in that class.	3.58 (SD = 0.44)	3.63 (SD = 0.67)	3.53 (SD = 0.66)
